# pH-Dependent Behavior of Novel 5-FU Delivery System in Environmental Conditions Comparable to the Gastro-Intestinal Tract

**DOI:** 10.3390/pharmaceutics15031011

**Published:** 2023-03-21

**Authors:** Geza Lazar, Fran Nekvapil, Branko Glamuzina, Tudor Tamaș, Lucian Barbu-Tudoran, Maria Suciu, Simona Cinta Pinzaru

**Affiliations:** 1 Biomolecular Physics Department, Faculty of Physics, Babes Bolyai University, Kogalniceanu 1, 400084 Cluj-Napoca, Romania; 2 Institute for Research, Development and Innovation in Applied Natural Science, Fântânele 30, 400327 Cluj-Napoca, Romania; 3 Department of Applied Ecology, University of Dubrovnik, Ćira Carića 4, 20 000 Dubrovnik, Croatia; 4 Department of Geology, Babeş-Bolyai University, 1 Kogălniceanu, 400084 Cluj-Napoca, Romania; 5 Electron Microscopy Centre, Babeș-Bolyai University, Clinicilor 5-7, 400006 Cluj-Napoca, Romania; 6 Advanced Research and Technology Center for Alternative Energy, National Institute for Research and Development of Isotopic and Molecular Technologies, Donat 67-103, 400293 Cluj-Napoca, Romania

**Keywords:** drug carrier, novel pharmaceutical formulation, pH-dependent release, SERS

## Abstract

A biogenic carrier for 5-fluorouracil (5-FU) loading and subsequent tableting as a new drug formulation for slow release has been proposed using the biomineral from blue crab carapace. Due to its highly ordered 3D porous nanoarchitecture, the biogenic carbonate carrier could achieve increased effectiveness in colorectal cancer cure provided that the formulation would successfully pass through the gastric acid conditions. Following the recently proven viability of the concept by demonstrating the slow release of the drug from the carrier using the highly sensitive SERS technique, here we investigated the 5-FU release from the composite tablet drug in pH conditions replicating the gastric environment. The released drug from the tablet was studied in solutions with three relevant pH values, pH 2, pH 3, and pH 4. The 5-FU SERS spectral signature for each pH value was used to build calibration curves for quantitative SERS analysis. The results suggested a similarly slow-releasing pattern in acid pH environments to that in neutral conditions. Although biogenic calcite dissolution was expected in acid conditions, the X-ray diffraction and Raman spectroscopy showed preservation of calcite mineral along with the monohydrocalcite during acid solution exposure for two hours. The total released amount in a time course of seven hours, however, was lower in acidic pH solutions, with a maximum fraction of ~40% of the total amount of loaded drug, for pH 2, as opposed to ~80% for neutral values. Nonetheless, these results clearly prove that the novel composite drug retains its slow-releasing character in environmental conditions compatible with the gastrointestinal pH and that it is a viable and biocompatible alternative for oral delivery of anticancer drug to reach the lower gastro-intestinal tract.

## 1. Introduction

In terms of mortality from cancer, colorectal cancer ranks third among both men and women, and is only surpassed by lung cancer [1]. The treatment methods for cancer are usually limited to chemotherapy, radiation, and surgery, which usually involve numerous side effects [2]. Because of its lack of invasiveness, simplicity, effectiveness, and higher rate of patient acceptance, the oral administration of therapeutic agents is the most desirable of these treatment methods [3,4,5,6,7]. Nonetheless, oral delivery presents several serious challenges that need to be overcome to achieve the desired results. Firstly, it is challenging to deliver the maximum therapeutic dose while also preventing the drug’s breakdown or absorption in the stomach and small intestine because the colon is the furthest region of the digestive system [8,9]. Moreover, sensitive pharmaceuticals may degrade faster in the stomach’s acidic pH conditions, while the small intestine’s and stomach’s proteolytic activities can cause the drugs to be denatured. The adsorption of the drug on a carrier followed by its slow release in the desired location appears to be the most viable solution to overcome these obstacles. The development of innovative drug delivery systems, for slow and controlled release, has been the focus of intensive research efforts over the past few years. Numerous types of drug carriers have been reported so far, such as mesoporous silica, biodegradable polymers, hydrogels, carbonaceous nanomaterials with biomaterials incorporated, carbon nanotubes, hydroxyapatite, or superparamagnetic nanoparticles, each with its own advantages and disadvantages [10,11,12,13,14,15,16,17,18,19,20,21,22,23,24,25].

In our previous work [26], we successfully developed a novel pharmaceutical formulation of 5-FU based on an abundant yet largely unused biomaterial, the wasted shells of the Atlantic Blue Crab (*Callinectes sapidus*). The biogenic waste material was chosen as drug carrier for 5-FU based on its remarkable physico-chemical properties, preparation method, and availability and biocompatibility, which confer considerable advantages over other drug delivery systems. We have previously shown that the shell of the Atlantic blue crab possesses an intricate, highly ordered three-dimensional (3D)-nanoarchitecture of a magnesian calcite (Mg-CaCO_3_) matrix with nanosized pores and channels and organic scaffolds comprising chitin–protein fibrils, rich in carotenoid pigments (astaxanthin), and caroteno-proteins [27,28]. The porosity and hierarchical 3D nanostructure of the biomaterial allow for powder preparations for solutions loading, adsorption, and subsequent active release of compounds, which are the key factors that enable the use of such biomaterial as successful carriers. 5-fluorouracil (5-FU) is one of the oldest anticancer drugs and it has been widely used in the treatment of cancer (such as colorectal, stomach, breast, brain, liver, and lung cancer). Although 5-FU is a popular drug that is extensively used in the treatment of colorectal cancer, alone or in combination with other drugs such as oxaliplatin and leucovorin [21], or as a precursor (for example, capecitabine chemotherapeutics), there are several disadvantages associated with its use, including rapid metabolism, short half-life low bioavailability, high cytotoxicity, or inadequate selectivity for cancerous cells, all of which severely limit the effectiveness of the drug [21,22,23,24]. To surmount these issues posed by the oral delivery 5-FU and enhance its effectiveness, lots of resources are devoted to the development of novel 5-FU delivery systems.

The biocompatibility of the biogenic CaCO_3_ is an additional advantage of the powder produced from the blue crab shell, which, combined with the antioxidant properties conferred by the carotenoids enclosed in the native biogenic material, not only significantly diminishes the risk of adverse side effects but could potentially confer further benefits against drug side effects. Astaxanthin is the major carotenoid natively embedded in crustacean shells either in free form or non-covalently bound in blue caroteno-proteins [27] from a biogenic scaffold. It has been demonstrated [29] that astaxanthin’s exhibited protective role in colon cancer is induced in animal models, although the involved mechanism still needs elucidation [29]. Moreover, a recent review [30] highlighted the astaxanthin anti-oxidant and anti-inflammatory effects against gastro-intestinal disorders including cancer.

The preservation of astaxanthin in biogenic powder derived from crustaceans is another issue that can be tracked during drug loading in carriers, tablets, and exposure to acidic conditions. Selective tracking of astaxanthin in carriers is achieved here by exploiting the resonance Raman technique, which allowed specific detection of this compound from a composite matrix under resonant the excitation of carotenoid, using the 532 nm laser line [27].

We previously conducted comprehensive analyses on the biogenic powders result33ing from crustacean shells [26,27,28]. Confocal Raman micro-spectroscopy (CRM), scanning electron microscopy (SEM), Brunauer–Emmett–Teller (BET) method [31], and X-ray diffraction (XRD) were used to investigate the morphology and composition of the blue crab shell powder pre [27,28] and post drug loading with pelleting into tablets [26], demonstrating the adsorption of the drug (5-FU) in the pores of the biogenic carrier [26]. Moreover, surface enhanced Raman scattering (SERS) was employed as a novel highly sensitive and specific method to monitor the slow release of the active ingredient (5-FU) from the newly formulated tablets suspended in water, thus quantifying the amount of drug released from the tablet and proving the concept and viability of the newly formulated composite drug [26].

The aim of the current work was two-fold, firstly to probe the powder derived from biogenic calcite from crustaceans as an effective drug carrier under acid pH conditions resembling the gastric environment, and secondly, to probe the sensitive SERS technique to quantify the released active ingredient under respective acid conditions compatible with the gastro-intestinal tract. For the validation of the results, complementary techniques, including X-ray diffraction, SEM-EDX, and NIR-Raman spectroscopy, were used to prove the identity of the tablet and active ingredient, as well as to determine the tablet morphology and the preservation of the biomineral before and after acid exposure.

## 2. Materials and Methods

### 2.1. Materials

5-Flourouracil powder with 99% purity (Merck, Darmstadt, Germany) was purchased from Nordic Chemicals (Cluj Napoca, Romania) and was used without further purification. A stock of shells from specimens of the Atlantic blue crab, *Callinectes sapidus*, fishing capture in the Parila Lagoon, Neretva river delta (Croatia; south–east Adriatic Sea) was employed as raw biogenic material. Caught medium-sized crabs (10–15 cm carapace width) were frozen at −20 °C until further preparation. Hydrochloric acid solutions with three pH values, 2, 3, and 4 were used in the slow-releasing experiments.

### 2.2. Shell Powder Preparation

The preparation of the biogenic shell powder is described in detail in our previous studies [26,27,28], and the same procedure was followed in the present paper.

### 2.3. Biogenic Powder and Particles Characterization

The experimental procedures employed for ball milling and pore size distribution analysis of the biogenic powder are detailed in our previous work [26], and the same procedures were followed in the present paper.

### 2.4. Tablets Preparation and Quantitative SERS Analysis of Released 5-FU

A solution of fluorouracil was prepared by dissolving 50 mg of 99% purity 5-FU powder in ultrapure distilled water (18.2 M ohm), resulting in a solution of 1 mg/mL concentration. This solution was used throughout the experiments. Five milliliters of 5-FU solution was been added to an identical volume of powdered blue crab carapace and milled for 5 min. The 5-FU solution was loaded in biogenic powder, which was previously characterized [26]. The resulting composite was dried at room temperature (20 °C), and the dried solid mass was mechanically pressed in tablets weighing between 300 and 600 mg using 3 tons of force press. The diameter of the resulting tablets was 10 mm.

Before and after acid exposure, the tablet surfaces were characterized using Raman micro-spectroscopy, and their mineral composition was checked using X-ray diffraction.

In a time series experiment, each tablet was individually suspended in an aqueous solution with pH adjustment, using HCl solutions to obtain three different pH values, 2, 3, and 4, at room temperature (20 °C). The solutions obtained from each suspended and stirred tablet were further tested using SERS spectroscopy for highly sensitive detection of any trace amount of released 5-FU. To enhance the Raman signal of a trace amount of 5-FU solution, a colloidal Ag surface was prepared according to the classic Lee–Meisel procedure [32]. The AgNPs used in the experiments were first tested on well-known SERS molecular species, such as methylene blue or 4-aminothiophenol [33,34], and are described in detail in our previous work [26].

The acid solutions with suspended tablets were continuously tested using SERS samples obtained by adding 10 μL of solution to 500 μL colloidal silver nanoparticles (AgNPs). SERS spectra were recorded three times for each aliquot, and SERS experiments were run in triplicate on all tablets in a time course from the first half hour of tablet suspension and further, at every hour, up to a total of 7 h of tablet suspension in a respective acid solution. Each SERS test on each tablet has been run under similar SERS sampling. Thus, the concentration of the released 5-FU could be determined by building a calibration curve of the SERS signal of 5-FU dissolved in water at the respective pH. Averaged SERS spectra from three distinct experiments were used to build the calibration curves.

### 2.5. Instruments and Data Processing

Confocal Raman spectra of tablets were acquired using a Renishaw (Wotton-under-Edge, UK) InVia Confocal Raman System and a Cobolt DPSS laser emitting at 532 nm, as well as a high-power NIR diode laser emitting at 785 nm.

Tablet surfaces were characterized before and after acid solution exposure using Raman spectroscopy with two excitation lines, one NIR line to achieve a general chemical characterization, and a 532 nm line to check the preservation of the native carotenoids in the biogenic carbonate. Raman spectra with 532 nm excitation achieving the resonance conditions for carotenoids allowed us to selectively detect these compounds. Running NIR-Raman with 785 nm excitation allowed us to identify the mineral components of the tablet before and after exposure to an aqueous solution in acid pH. During Raman microscopy, 5× (NA 0.12 WD 13.2 mm), 20× (NA 0.35, WD 2 mm) and 100× (NA 0.9, WD 3.4 mm) collecting objectives were used with theoretical spatial resolutions of 2.7 μm, 0.927 μm, and 0.36 μm, respectively. An edge filter was employed to record spectra in the 90–1840 cm^−1^ spectral range with 0.5 cm^−1^ resolution and with the signal detected using a Rencam CCD. The acquisition parameters used in the Raman measurements of tablets were 1 s exposure time, 1 accumulation with a 200 mW laser power for the 532 laser line, and 300 mW laser power for the 785 nm line, respectively. The SERS spectra of solutions were acquired using a fast, portable, i-Raman Plus B&W TEK spectrometer, equipped with a 532 nm laser line. The acquisition parameters used in the SERS experiments, on liquid samples, were 30 s exposure time, and 10 acquisitions with 320 mW output laser power. Data processing has been achieved with OriginPro 8.5 software (OriginLab, Northampton, MA, USA)

Scanning electron microscopy (SEM) imaging of the tablets was conducted on a Hitachi SU8230 cold-field emission electron microscope; more experimental details can be found in our previous work [26].

The mineral composition of the tablets was probed by X-ray diffraction (XRD) using a Bruker D8 Advance diffractometer (Bruker, Billerica, MA, USA); the procedure is detailed in our previous work [26].

### 2.6. Data Analysis

SERS measurements were run on aqueous solutions containing 5-FU in various concentrations, at each pH value, in order to build calibration curves of the SERS spectral intensity as a function of concentration. These acquisitions were performed for each concentration, and the resulting averaged SERS spectra for each concentration were used to generate the calibration curves. The curves were fit using linear models, resulting in the following coefficients of determination: R^2^ = 0.96 for pH 2, R^2^ = 0.863 for pH 3, and R^2^ = 0.971 for pH 4.

Similarly, 3 SERS spectra were recorded for each solution obtained from the suspended tablet at all the studied time intervals of the release experiment. The SERS spectra were averaged, and the released amount was quantified using the previously mentioned calibration curve. The results were subsequently plotted as a function of time, and the release curve was fitted following an allometric growth model. The coefficients of determination were as follows: R^2^ = 0.83 for pH 2, R^2^ = 0.919 for pH 3, and R^2^ = 0.854 for pH 4.

## 3. Results and Discussion

**Figure 1:**
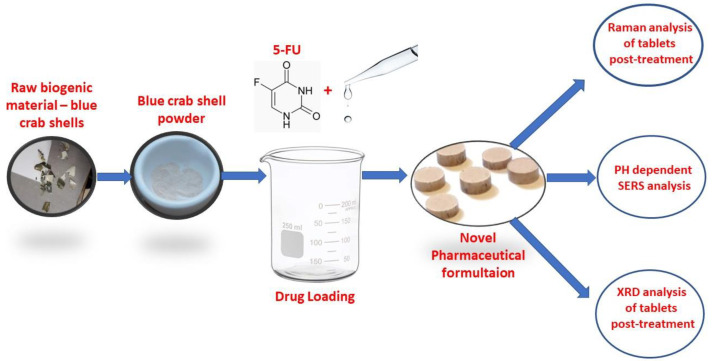
Schematic design of the biogenic calcite drug carrier for loading the 5-FU and the pH effect on the tablet and its slow-releasing active ingredient.

Figure 1 presents the experimental procedure followed in the preparation of the novel composite drug tablets, followed by their characterization and further investigation of the 5-FU slow release in acidic solutions compatible with the gastro-intestinal (GI) tract solution. The ultrasensitive surface enhanced raman scattering (SERS) technique was used for 5-FU detection, as well as scanning electron microscopy (SEM) and X-ray diffraction (XRD) to investigate the effects of acid exposure on the composite tablets.

**Figure 2:**
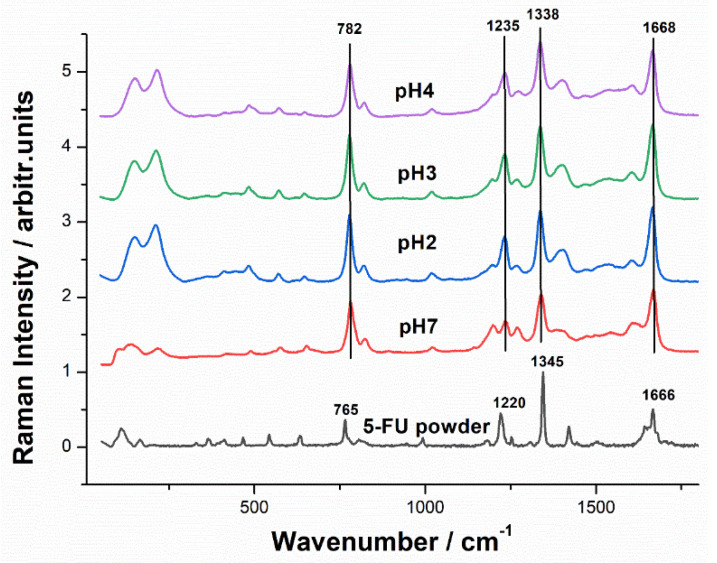
Micro-Raman spectrum of 5-FU powder (lower) compared to the SERS signal of the 5-FU solution (0.02 μg/mL) on AgNPs at various pH values: pH 7 (ultrapure water), pH 4, pH 3, and pH 2. Raman and SERS spectra were acquired using 1 s exposure time, 1 acquisition, 220 mW laser power, and a 20× magnification microscope objective. Excitation: 532 nm.

Ball milling was used to grind down the biogenic crab shell to a fine powder, in order to be loaded with 5-FU. The particle size distribution of the powder resulting after 3 min of milling is similar to the previous batch described in Figure 2 from our previous paper [26], therefore not repeated here. The powder presented BET surface areas below 10 m^2^/g (7.17 m^2^/g) with a pore volume of 0.014 cm^3^/g. The pore volume and size distribution of the blue crab shell powder were studied and described in detail in our previous papers [26,27,35] and therefore are not given here.

The above-mentioned powders were loaded with 5-FU solution and after drying were pressed into tablets. Their spectroscopic characterization using confocal Raman micro-spectroscopy showed that the calcium carbonate structure as well as the minor organic components (mainly caroteno-proteins, chitin, and free carotenoids) were retained and that no traces of 5-FU signal could be found on the surface, proving that the drug was adsorbed in the nanopores. The typical Raman signal of the native blue crab shell fragments excited with a near-infrared laser line (785 or 1064 nm) revealed calcium carbonate Raman bands along with weaker bands from chitin, astaxanthin carotenoid pigment, and caroteno-proteins spectral signature [27]. Powdering the material, Raman spectroscopy is seriously hampered by the increased fluorescence due to the larger exposure of the organic scaffold. However, Raman spectra of powders still revealed the carotenoid preservation when spectra were excited in resonance conditions for these compounds (532 nm) and the characteristic bands of calcite mineral when a NIR laser line was used for excitation. In both cases, the signal was observed on a high spectral background. To distinguish the two different components in tablets, two excitation wavelengths were used—a laser line emitting at 532 nm to resonantly excite the carotenoids, and the near-infrared line emitting at 785 nm—nonresonantly provided the overall signature of all the molecular compounds from sample. The signature of the carotenoids in the biogenic material was identified by the two main Raman bands at 1153 and 1516 cm^−1^, assigned to the characteristic C−C and C=C bonds of the skeletal chains in astaxanthin, while the calcium carbonate presence was confirmed by the main stretching mode of the carbonate at 1083 cm^−1^. The Raman signal collected from the surface of the tablets was consistent with the characteristic signal of the blue crab powder and with no trace of the 5-FU signature, proving that the morphological properties of the material were retained after pelleting and that the drug was adsorbed into the nanopores, in agreement with the previous study of similar tablets [26].

The characteristic Raman spectra of the 5-FU powder and the SERS signal of the 5-FU aqueous solution at different pH values are presented in Figure 2. The most intense SERS bands of fluorouracil appear at 786, 1234, 1334, and 1667 cm^−1^, respectively, and are assigned to the pyrimidine ring breathing, the complex mode comprising ring + C−F wagging, ring + C−H wagging modes, and the symmetrical stretching of the C-O bond, respectively [36,37]. The position and intensity of the bands are in close correlation with the pH and the concentration of the solution.

To study the slow release of fluorouracil from the nanopores of the pellets in environmental conditions compatible with the GI tract, the pellets were suspended in aqueous solutions with 3 relevant acid pH values: pH 2, pH 3, and pH 4. Prior to the release experiments, however, it was necessary to investigate the variation of the 5-FU SERS signal as a function of concentration, for each pH value, to be able to accurately quantify the released amount.

### 3.1. Quantitative SERS Analysis of 5-FU in Aqueous Solutions at Acid pH

**Figure 3:**
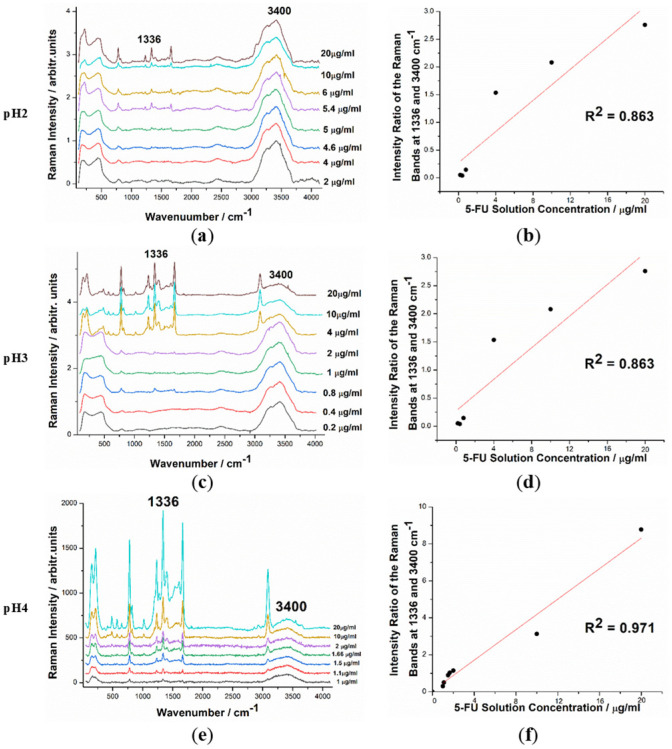
Normalized SERS spectra of 5-FU at concentrations ranging from 0.2 to 20 μg/mL, with the main SERS bands tagged for pH 2 (**a**), pH 3 (**c**), and pH 4 (**e**). Intensity ratio of the 5-FU band at 1336 cm^−1^ and the water band at 3400 cm^−1^ plot against the concentration for pH 2 (**b**), pH 3 (**d**), and pH 4 (**f**).

For this purpose, solutions with concentrations ranging from 0.2 to 20 μg/mL were prepared for each pH value. SERS spectra were recorded and analyzed for each solution to determine the dependence of the SERS signal on the 5-FU concentration. Using the intensity of one of the main 5-FU bands at 1338 cm^−1^ relative to the intensity of the water band at 3400 cm^−1^, we were able to build reliable calibration curves for each of the solutions with varying acidities. The SERS spectra of the samples, along with the calibration curves, are presented in Figure 3. As expected, the SERS intensity of the 5-FU band decreases as the solutions are diluted. The calibration curves were linearly fitted for each of the three cases studied with the R^2^ (coefficient of determination) values of 0.96, 0.86, and 0.97 for pH 2, 3, and 4, respectively. The resulting fitted curves were used as references in further experiments on solutions from suspended tablets in acidic pH conditions and calculations.

### 3.2. Composition and Morphology of the Tablets Exposed to Acidic pH Conditions

**Figure 4:**
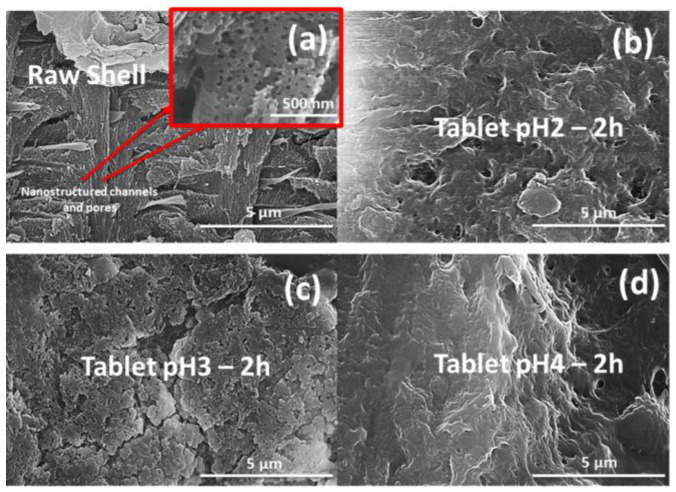
Scanning electron microscopy (SEM) image of the raw biogenic fragments of blue crab shells (**a**) compared to the tablet exposed to acidic pH: pH 2 (**b**), pH 3 (**c**), and pH 4 (**d**).

Besides studying the slow release of the drug from the pellets in acidic conditions, we were interested in determining the effects of acid exposure on the composite tablets using acid solutions with pH values similar to the solutions used in the release experiments (pH 2, pH 3, and pH 4). After 2 h of exposure, the tablets were removed from the acidic solutions and were studied using scanning electron microscopy (SEM), Raman spectroscopy, and X-ray diffraction. In Figure 4, the scanning electron microscopy (SEM) images of the surface of the treated pellets compared to the surface of the raw biomaterial are presented. In the top left image (raw shell), the highly ordered 3D nanostructure of channels and pores is clearly visible. However, following exposure to acidic solutions, the images of the tablet surfaces seemingly suggest that the 3D nanoarchitecture was slightly damaged. The nanochannels and pores are not visible anymore; instead, we can observe sizeable holes and cracks in the structure. Moreover, as expected, the most affected sample seems to be the tablet exposed to the solution with pH2 showing the largest and the most holes. The SEM images suggest that although the calcium carbonate crystalline structure was not affected by the acid exposure, and the organic matrix, crucial to the porous structure, was damaged to a certain extent.

**Figure 5:**
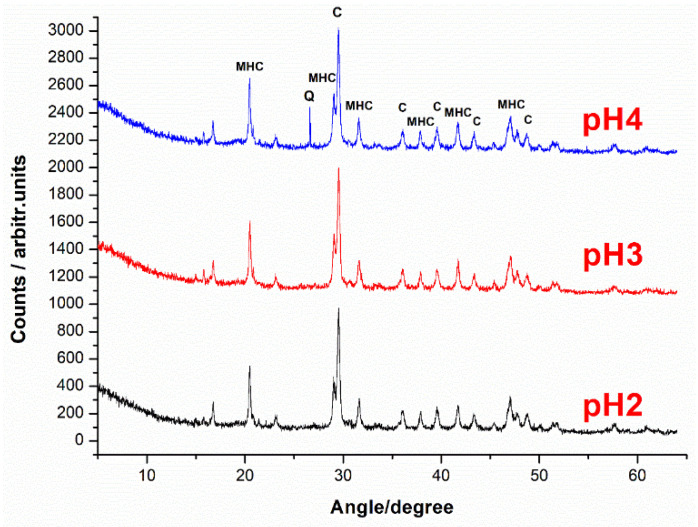
XRD pattern of the tablets exposed to acidic pH revealing the crystalline calcite signal (marked with C on the graph), as well as the monohydrocalcite signal (marked with MHC) and traces of quartz, probably from embedded environmental sand grain in native shell roughness.

Figure 5 presents the XRD patterns of the tablets exposed to acidic solutions. The data reveal the coexistence of the crystalline calcium carbonate as well as monohydrocalcite. Traces of quartz signal were spuriously observed (denoted Q in Figure 5), probably from environmental sand traces not completely washed in raw shell fragments. The characteristic peaks of monohydrocalcite (MHC) are located at 20.5, 29. 04, 31.5, 37.81, 39.59, 41.75, and 47.11 degrees [38] and were used for identification in tablet samples. The crystalline calcium carbonate C was identified by its characteristic peaks at 29.5, 36.1, and 39.6 degrees [38]. The presence of MHC is somewhat expected in light of our previous study [35] due to the contact of amorphous calcium carbonate counterpart from the native shell (smaller fraction than the main calcite) with water, during powder loading with an aqueous solution of the drug.

**Figure 6:**
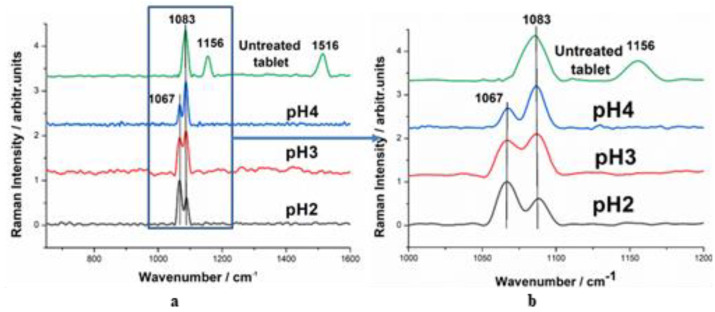
Confocal Raman spectra of the tablets exposed to acidic pH revealing a crystalline calcium carbonate signal as well as the monohydrocalcite signal, on the 700–1600 cm-1 spectral range (**a**) and 1000–1200 cm-1 spectral range (**b**).

Raman spectra of tablets after 2 h immersion in aqueous solutions at acid pH are presented in Figure 6. Two prominent bands, one from calcite (1083 cm^−1^) and one from MHC (1067 cm^−1^) [39], were observed either in neutral conditions or in acid solutions. It is important to notice the change in the relative intensity of the MHC and calcite bands for the different pH exposure values. Seemingly, the intensity of the 1083 cm^−1^ band is decreasing as the strength of the acid solution increases. This suggests that although the crystalline structure of the tablet is retained, the exposure to acid does affect the mineral composition and crystallinity of the tablet surface. Furthermore, the specific carotenoid signal marked by the intense bands at 1156 and 1516 cm^−1^ cannot be noticed after acid treatment, confirming that the organic scaffold was damaged by exposure to acidic solutions. More in-depth studies are necessary, however, to accurately determine the extent of these effects. The extraction of carotenoids in an acid solution is less plausible since their signal was not detected in the SERS analysis.

**Figure 7:**
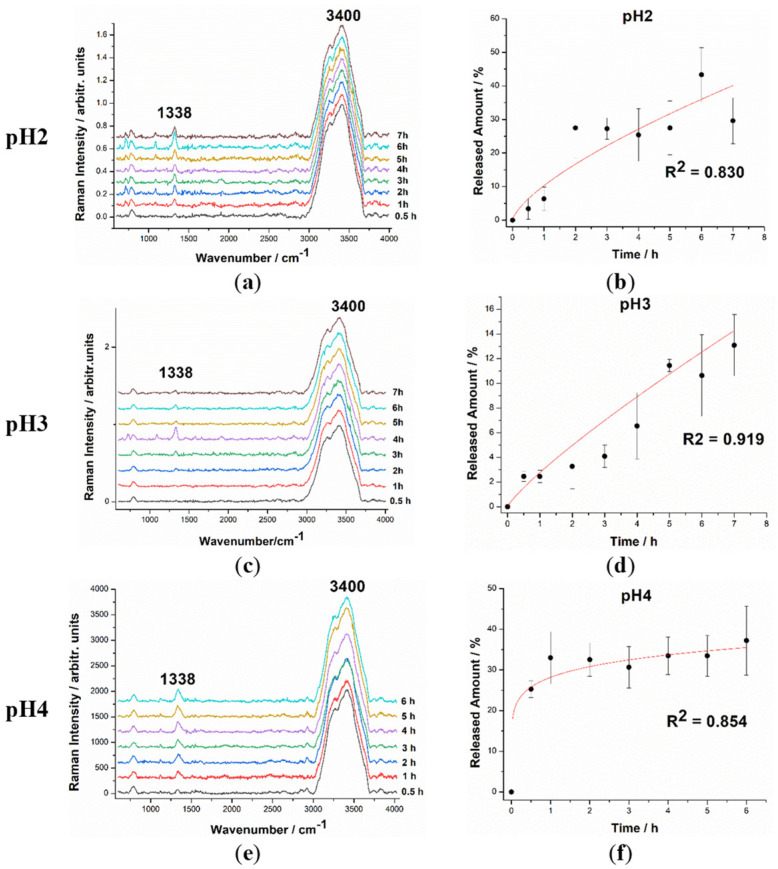
Averaged SERS spectra of the samples collected from the solutions containing the tablet over several hours of experiment for each pH value: pH 2 (**a**), pH3 (**c**), pH 4 (**e**). Released amount of FU from the tablet as a function of time for each pH value: pH 2 (**b**), pH3 (**d**), and pH 4 (**f**).

With the goal of verifying the viability of the novel composite drug in environmental conditions compatible with the GI tract, the slow release of the drug in acidic solutions was investigated. By diluting a 1% HCl stock solution in ultrapure distilled water, three solutions with pH 2, 3, and 4 were used. Three tablets loaded with 5-FU with masses ranging from 270 to 600 mg were suspended in these solutions at room temperature (20 °C) and used for three time-series SERS experiments over the course of 7 h, with measurements acquired after half an hour for tablet suspension and hourly afterward. For each measurement, 10 µL of the solution was collected and mixed with 500 µL of AgNPs colloidal solution, with at least three measurements performed for each sample and the obtained spectra subsequently averaged. The averaged SERS spectra acquired at each time interval for each pH value are shown in Figure 7a,c,e. As previously mentioned for the quantification of the released amount, we used the intensity of the characteristic 5-FU band at 1338 cm^−1^ relative to the intensity of the water band at 3400 cm^−1^. The 1338 cm^−1^ band is clearly visible after 0.5 h both in the case of the pH 2 solution as well as in the case of the pH 4 solution, while in the case of the pH 3 solution, the peak intensity was extremely low but still identifiable. This suggests that the start of the release process is faster in acidic conditions when compared to our previous experiments performed in distilled water, where the released 5-FU could only be detected starting from the second hour of the experiment (this could also be caused by lower detection limits). As the experiment progressed over time, the 1338 cm^−1^ band became clearly visible in each spectrum with increasing intensities. Using the previously presented calibration curves for each pH value for the pure 5-FU solution as references, the variation in the relative intensities of the 1338 cm^−1^ and 3400 cm^−1^ bands allowed the quantification of the amount of 5-FU released from the tablets as a function of time. The intensity ratio was determined for each measurement, the results were averaged, and the relative errors were calculated for each sample. The amounts released were then plotted against time, with the released 5-FU given as a percentage of the total amount of drug contained in the tablet.

The resulting graphs (Released Amount vs. Time) for each pH value can be seen in Figure 7b,d,f. The experiments were performed over a similar time span (6 and 7 h) as in our previous work, to be able to compare the release pattern in acidic solutions to the pattern in water. As previously stated, the releasing process began from the first hour of the tablet suspension and continued up to the 6th and 7th hours. From the tablets suspended in solutions of pH 2 and pH 4, the release seemed to slow down after the first 2 h of the experiment with ~30% of the total amount released after 2 h, but only 40% after 6 and 7 h, respectively. For the tablet suspended in pH 3 solution, the release pattern was slightly different, with the released amount having a more linear trend over the course of time. Although the data are plotted as means (*n* = 3), the results are subject to significantly larger relative errors, which might be due to complex factors related to the calcium carbonate polymorphs (calcite and amorphous) behavior, the slow formation of MHC when the fraction of amorphous calcium carbonate coexisted in the presence of water, factors related to the AgNPs efficiency for SERS signal enhancement in complex solutions comprising multiple anions and cations, and related to the molecular behavior of 5-FU released in such complex aquatic media. Such influences include the dynamics of the CO_2_ expected to occur in aqueous solution at acidic pH values, as a result of calcium carbonate reaction in acid conditions, the co-existence of Ca^2+^ ions in solution and their slight influence on the AgNPs aggregation [40], subsequent 5-FU SERS intensity output, and the co-existence of CO_3_^2−^/HCO^3−^ in solution. The Fermi diads of solvated CO_2_ in Raman spectra are known to occur at about 1278 and 1386 cm^−1^. These peaks were not observed in SERS spectra of the solution from he suspended tablet, indicating that such a reaction is too weak to be detectable or too fast, and the carbon dioxide was eliminated as a bubbling gas from the test solution. Nevertheless, these complex phenomena still need additional dedicated studies to be fully understood.

It is important to note that the eventual protonation of the 5-FU in an acid environment immediately after their release does not affect the chosen SERS band intensity to quantify the 5-FU amount. The total released amount after 7 h added up to ~40% of the total amount of drug contained in the tablets, in the case of the pH 2 and pH 4 solutions; for the pH 3 solution, this value was significantly lower at only ~15%. Nonetheless, each graph was fitted following allometric growth models with relatively high CODs (0.8; 0.92 and 0.93), the same model used to fit the release curve in distilled water, showing that the release patterns are similar regardless of the pH of the solution. It is notable, however, that the total released amount in acidic conditions is significantly lower than the amount released in neutral water at ~80%. This difference in the released amount between acid and neutral conditions is likely caused by the chemical reactions occurring at the surface of the tablets when exposed to acid. Both the XRD and the SEM results point toward a slight deterioration of the tablet surface caused by the acid environment, which could damage the porous nanostructure and thus slow down or even shut down the release process. In practice, though, the fact that only 40% of the active ingredient is released is only a minor hurdle, as the dosage is easily adjustable according to the specific needs.

Nevertheless, the present results clearly demonstrate that the composite tablets retain their release properties when placed in acidic conditions, regardless of the biological pH values.

## 4. Conclusions

Advancing from our previous works regarding the proof of concept in using biogenic powders for anticancer drug delivery formulation, we have investigated and demonstrated here the huge potential of the biogenic calcium carbonate originating from blue crab to be used as a drug carrier in biomedical applications in relevant pH environments. The current study seeks to further solidify our previous findings and prove the viability of the biogenic material. Although expected to record large differences regarding the tablets’ behavior in neutral aqueous solutions compared to acid exposure, the results here clearly showed that this novel pharmaceutical formulation retains its properties at low pH values, comparable with acid conditions from the digestive tract in biological organisms. The fact that the slow release of the drug from the tablet is maintained in acidic conditions is crucial, as it ensures that the previously mentioned cytotoxic side effects are greatly diminished. Although the tablets released only ~40% of the 5-FU they were loaded with, at low pH conditions, this is only a minor obstacle, as the size of the tablets and dosage can be adjusted according to the specific needs. Moreover, in spite of the fact that the results suggest the deterioration of the tablet surface following acid exposure, the slow-releasing process is hardly affected by this superficial modification. Thus, we can confidently state that blue-crab-shell-based composite drug is a viable, advantageous, and attractive alternative for oral administration of 5-FU loaded in biogenic ultrastructed powder for reaching the lower digestive tract as an improved cancer treatment.

In comparison with other formulations, the use of blue crab shells as biogenic carriers presents numerous advantages. Most importantly, owing to its calcium carbonate backbone, the biomaterial is biocompatible, completely eliminating the risk of adverse side effects. Even more, its carotenoid content, which possesses antioxidant properties, could bring further benefits to the organism. In terms of practicability, this method also has advantages over other formulations; the loading process is relatively simple and the raw biomaterial needs minimal physical preparation prior to drug loading, and it does not require any complex or expensive equipment, making the method easily scalable for industrial use. Moreover, the crab shell is an accessible waste material, and its use falls in line with the concepts of the circular economy and blue bioeconomy.

## Data Availability

The data are available on request from the first or corresponding author.
